# Microvascular Blood Flow Improvement in Hyperglycemic Obese Adult Patients by Hypocaloric Diet

**Published:** 2016-11-01

**Authors:** T Mastantuono, M. Di Maro, M. Chiurazzi, L. Battiloro, N. Starita, G. Nasti, D. Lapi, L. Iuppariello, M. Cesarelli, G. D’Addio, A. Colantuoni

**Affiliations:** 1 Department of Clinical Medicine and Surgery, “Federico II” University Medical School, Naples, Italy; 2 Department of Biomedical, Electronics and TLC Engineering, University of Naples, “Federico II”, Naples, Italy; 3 IRCCS S. Maugeri Foundation, Telese, Benevento, Italy

**Keywords:** obesity, hyperglycemia, hypocaloric diet, microvascular blood flow, laser Doppler perfusion monitoring

## Abstract

The present study was aimed to assess the changes in skin microvascular blood flow (SBF) in newly diagnosed hyperglycemic obese subjects, administered with hypocaloric diet. Adult patients were recruited and divided in three groups: NW group (n=54), NG (n=54) and HG (n=54) groups were constituted by normal weight, normoglycemic and hyperglycemic obese subjects, respectively. SBF was measured by laser Doppler perfusion monitoring technique and oscillations in blood flow were analyzed by spectral methods under baseline conditions, at 3 and 6 months of dietary treatment. Under resting conditions, SBF was lower in HG group than in NG and NW ones. Moreover, all subjects showed blood flow oscillations with several frequency components. In particular, hyperglycemic obese patients revealed lower spectral density in myogenic-related component than normoglycemic obese and normal weight ones. Moreover, post-occlusive reactive hyperemia (PORH) was impaired in hyperglycemic obese compared to normoglycemic and normal weigh subjects. After hypocaloric diet, in hyperglycemic obese patients there was an improvement in SBF accompanied by recovery in myogenic-related oscillations and arteriolar responses during PORH. In conclusion, hyperglycemia markedly affected peripheral microvascular function; hypocaloric diet ameliorated tissue blood flow.

## I. INTRODUCTION

Hyperglycemia is known as the first sign of glucose metabolic impairment, affecting several organ functions [1]. Hyperglycemia, indeed, contributes to micro-angiopathy, causing target-organ damage such as retinopathy and nephropathy in diabetes [2–5]. Therefore, it is important to determine the early dysfunctions, such as microvascular impairment, leading to severe complications.

Laser Doppler perfusion monitoring (LDPM) is a non-invasive and real-time diagnostic method to evaluate microvascular function [6–8]. This technique permits to study human skin blood flow under several pathophysiological conditions [9–13]. The skin represents, indeed, an easily accessible organ and the skin microvascular dysfunctions have been suggested to be related to microvascular changes in different organs [14]. Moreover, spectral analysis of LDPM signals allows us to accurately evaluate different mechanisms regulating microcirculation. In particular, data derived from human skin, reveal rhythmic oscillations in the frequency interval 0.005–2.0 Hz. These fluctuations in blood flow have been demonstrated to be associated with several physiological factors, involved in the regulation of blood flow: the NO-independent and NO-dependent endothelial activities, the sympathetic nervous system discharge, the intrinsic myogenic activity of vascular smooth muscle cells, the respiration and the heart rate [15–17].

Therefore, the aim of present study was to investigate the microvascular impairments in newly diagnosed hyperglycemic obese subjects using noninvasive LDPM technique. Skin microvascular blood flow (SBF) was evaluated under resting conditions in all recruited patients. Moreover, to assess peripheral microvascular reactivity, post-occlusive reactive hyperemia (PORH) was evaluated in a smaller group of patients. Oscillations in blood flow were analyzed by spectral analysis of LDPM signals to study the several factors modulating the blood distribution [18]. Finally, the effects of a low-glycemic and hypocaloric diet, administered for 6 months, were investigated in hyperglycemic subjects. This group was compared with an age-matched group of normoglycemic obese and an age-matched group of normal weight subjects.

## II. METHODS

### Study groups

Fifty-four normoglycemic obese subjects (27 females, NG group); fifty-four newly diagnosed hyperglycemic obese patients (27 females, HG group) and fifty-four normal weight subjects (27 females, NW group) were recruited from the Outpatient Clinic of the Department of Clinical Medicine and Surgery, “Federico II” University of Naples. In the HG group, hyperglycemia was diagnosed within 2 months before the study.

All patients were 55–64 years old; those belonging to NG and HG groups were obese [body mass index (BMI) ≥ 30 Kg/m^2^], while those of NW group were normal weight (BMI between 19 – 25 Kg/m^2^). Exclusion criteria were: presence of disease influencing body composition (cancer, osteoporosis and muscular dystrophia), heart failure and microangiopathy; treatment with drug influencing microvascular blood flow (such as vasodilators and anti-inflammatory drugs), insulin or oral hypoglycemic drugs and smoking.

The study was approved by “Federico II” University Institutional Ethical Committee; all patients gave their informed consent at recruitment.

### Dietary treatment

A hypocaloric diet (25 kcal/kg body desirable weight), with 55–60 % of total caloric intake in carbohydrates, 10–15 % in proteins and 25–30 % in fatty acids (< 7% saturated fats), was recommended to all obese patients. Carbohydrates were chosen according to their low glycemic index, while mono- and disaccharides were less than 7% of the total carbohydrate intake. NW group did not change the daily usual diet.

### Study protocol

Under baseline conditions (time T_0_), at 3 (time T_1_) and 6 (time T_2_) months of low-glycemic and hypocaloric diet administration, nutritional status, skin microvascular blood flow and blood flow oscillations were investigated. The NW group was studied at T_0_ and T_2_.

Nutritional status was evaluated by anthropometric measurements: weight, body mass index (BMI), waist circumference (WC), hip circumference (HC) and triceps skinfold (TS). Bioimpedance analysis (Akern RJL, BIA 101) was carried out to evaluate Fat Mass (FM) and Fat Free Mass (FFM). Glycemia (Gly), glycated hemoglobin (HbA1c) and basal insulin were measured. Moreover, the insulin resistance was estimated by the homeostasis model assessment of insulin resistance (HOMA-IR).

Microvascular blood flow evaluation was performed on the patients in supine position in a quiet and temperature-controlled room (22 ± 23 °C). No subjects had any medication, food, alcohol and/or drinks containing caffeine 12 h prior to the blood flow measurement. SBF was recorded using a laser Doppler perfusion monitoring apparatus (PeriFlux 4001 System, Perimed, Stockholm, Sweden) with the following characteristics: 780 nm wavelength, 10 Hz – 9 kHz bandwidth, 0.02 s time constant, 32 Hz sampling frequency. The LDPM apparatus was connected with a probe (PF 408, Perimed, Stockholm, Sweden), placed on the right forearm volar surface, and with a computer. After 10 min of acclimatization period, digital blood flow was recorded for 20 min by a Perisoft software. The mean value of SBF was expressed as arbitrary perfusion units (PU), while the power spectral density (PSD) of laser Doppler signals was reported as PU^2^/Hz. Finally, skin blood flow oscillations were analyzed by Wavelet transform [18].

Post-occlusive reactive hyperemia was evaluated in 14 normoglycemic obese subjects (7 females), 16 hyperglycemic obese patients (8 females) and 15 normal weight persons. After 20 min recording of microvascular blood flow under resting conditions, the brachial artery was occluded by a blood pressure cuff, placed at the right upper arm and inflated up to 50 mmHg above the systolic blood pressure. The blood pressure cuff was sudden deflated after 2 min brachial artery occlusion. Peak value (PK) was determined in PU as maximal perfusion value during hyperemia; percent increase of peak value (PK%) was calculated from the basal mean value; time to peak (Tp) was measured in seconds as the time from cuff release to the peak value; duration of hyperemia was evaluated in seconds as the time from cuff release up to the recovery of the mean value.

All data were expressed as mean ± standard error of the mean (SEM). Data were tested for normal distribution with the Kolmogorov-Smirnov test. Parametric (Student’s t tests, ANOVA and Bonferroni post hoc test) or nonparametric tests (Wilcoxon, Mann-Whitney and Kruskal-Wallis tests) were used. The statistical analysis was carried out by SPSS 14.0 statistical package. Statistical significance was set at p<0.05.

### Spectral analysis

Microvascular blood flow oscillations, in the range: 0.005 to 2.0 Hz, were evaluated by Wavelet transform, a scale dependent method comprising an adjustable window length able to analyze both low and high frequencies [18]. Spectral analysis was performed on 20 min recordings under resting conditions, to obtain higher resolution of very low frequency components. Wavelet analysis, proposed by Morlet, permits to detect at least six frequency components in this interval, as reported by Stefanovska et al [15, 16]. At first the overall spectral density of each frequency interval was determined; then the normalized spectral density for each frequency interval was evaluated as the ratio between the average spectral density of a specific frequency interval and the average total power spectral density. Therefore, the relative contribution of each frequency component was defined for the entire spectrum.

## III. RESULTS

The anthropometric measurements and the metabolic parameters of all recruited subjects are reported in Tables 1, 2 and 3. Under baseline conditions, all obese patients showed changes in the nutritional status with central obesity. In addition, hyperglycemic patients had significant higher levels of Hb1Ac, basal insulin and HOMA index compared to normoglycemic and normal weight ones. At 3 and 6 months of low-glycemic and hypocaloric diet, all obese patients showed a significant reduction in BMI and anthropometric parameters, accompanied by an improvement in body composition: fat mass was significantly reduced in kilograms and in percentage, while percent fat free mass significantly increased. Furthermore, glycemia, Hb1Ac, basal insulin and HOMA index significantly improved in hyperglycemic patients after dietary treatment (Table 1 and 2).

### SBF evaluation under resting conditions

**Figure 1:**
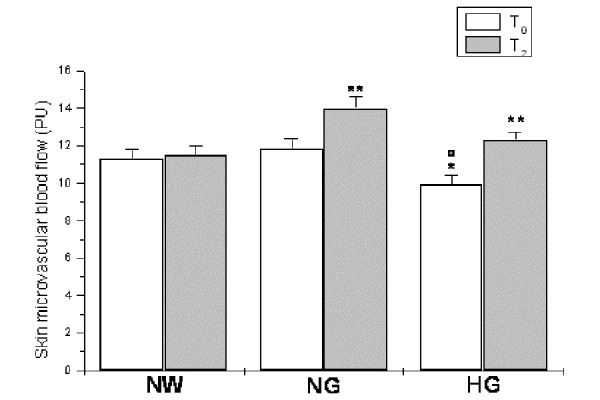
Skin microvascular blood flow, expressed as Perfusion Units (PU), under baseline conditions (T_0_) and at six months (T_2_) of diet administration in normal weight (NW), normoglycemic (NG) and hyperglycemic patients (HG). ^∘^ p<0.01 vs T_0_ NW group, * p<0.01 vs T_0_ NG group ; ** p< 0.01 vs T_0_

LDPM measurements revealed significant differences between normoglycemic and hyperglycemic obese patients under baseline conditions (time T_0_) compared to normal weight subjects. Hyperglycemic subjects showed lower mean value of SBF than normoglycemic ones (9.9 ± 0.5 vs 11.8 ± 0.6 PU, p<0.01 vs NG group; Figure 1), while normal weight subjects had baseline perfusion of 11.3 ± 0.5 PU (p<0.01 vs HG group). Moreover, spectral analysis indicates that total PSD of SBF recording was lower in the hyperglycemic than in normoglycemic obese and normal weight subjects (113.2 ± 10.1 vs 176.5 ± 11.4 and 170.4 ± 12.3 PU^2^/Hz, p<0.01 vs NG and NW group).

**Figure 2:**
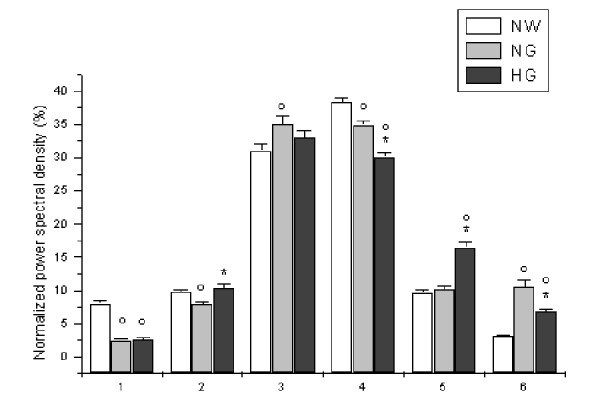
Normalized power spectral density, expressed as percent, related to NO-independent endothelial activity (1), NO-dependent endothelial activity (2), neurogenic activity (3), myogenic activity (4), respiration (5) and heart rate (6) under baseline conditions (T_0_) in normal weight (NW), normoglycemic (NG) and hyperglycemic patients (HG). ^∘^ p< 0.01 vs T_0_ NW group, * p< 0.01 vs T_0_ NG group.

Under baseline conditions, in all LDPM tracings, several frequency components were detected; four in low frequency component ranges: 0.005–0.0095 Hz, 0.0095–0.021 Hz, 0.021–0.052 Hz and 0.052–0.15 Hz. These frequency components were related to the NO-independent and NO-dependent endothelium activity, neurogenic and myogenic activities, respectively. Other two high frequency components were detected in the ranges: 0.15–0.6 Hz and 0.6–2.0 Hz, related to the respiratory and heart rates, respectively. The power spectral density of neurogenic and myogenic activities were higher than those related to the other parameters, as observed in normal weight subjects (Figure 2). However, in hyperglycemic patients the PSD related to myogenic activity (in percentage of total spectral density) was significantly lower than in normoglycemic obese and normal weight subjects (30.1 ± 0.7 vs 34.9 ± 0.7 and 38.3 ± 0.6 %, p<0.01 vs NG and NW groups; Figure 2). On the other hand, percent NO-dependent spectral density was higher (10.3 ± 0.7 vs 7.9 ± 0.3 %, p<0.01 vs NG) in hyperglycemic than in normoglycemic obese patients, but there was no significant difference when compared with the values evaluated in normal weight subjects (9.8 ± 1.0 %; Figure 2). The changes in the low frequency component spectral density were accompanied by the corresponding variations in high frequency components: respiration-related spectral density was higher in hyperglycemic than in normoglycemic and normal weight persons (16.5 ± 0.8 vs 10.1 ± 0.6 and 9.7 ± 0.5 %, p<0.01 vs NG and NW groups), while hearth rate related spectral density was the lowest in normal weight subjects (3.1 ± 0.2 vs 10.5 ± 1.0 and 6.8 ± 0.4 %, p<0.01 vs NG and HG groups) (Figure 2).

**Figure 3:**
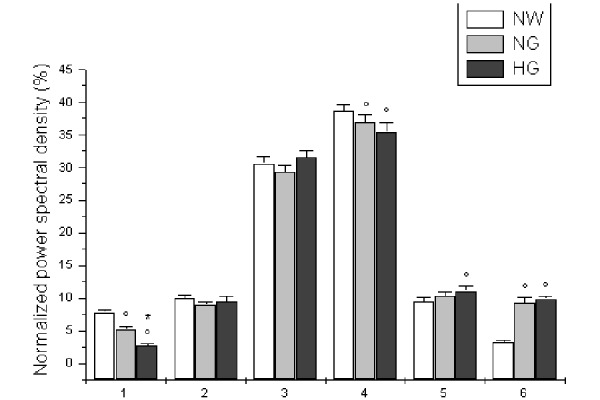
Normalized power spectral density, expressed as percent, related to NO-independent endothelial activity (1), NO-dependent endothelial activity (2), neurogenic activity (3), myogenic activity (4), respiration (5) and heart rate (6), at six months (T_2_) of diet administration in normal weight (NW), normoglycemic (NG) and hyperglycemic patients (HG). ^∘^ p< 0.01 vs T_2_ NW group, * p< 0.01 vs T_2_ NG group.

At 6 months of dietary treatment (time T_2_), SBF (Figure 1) and total PSD of LDPM oscillations significantly increased in hyperglycemic patients. In particular, at 6 months of a low-glycemic and hypocaloric diet administration, SBF was 12.3 ± 0.4 PU (p<0.01 vs T_0_; Figure 1), while total PSD of LDPM signals was 323.3 ± 10.1 PU^2^/Hz (p<0.01 vs T_0_). Furthermore, spectral analysis revealed a significant increase in myogenic related component (35.4 ± 1.5 vs 30.1 ± 0.7 %, p<0.01 vs T_0_), while NO-dependent endothelial-related component showed a trend to decrease (9.4 ± 0.9 vs 10.3 ± 0.7) (Figure 3). Finally, high frequency components showed a decrease for the respiration and an increase for heart rate-related components, compared to T_0_ values (p<0.01). In normal weight subjects, we did not detect any significant parameter change, at 6 months of observation (Figure 3).

### SBF evaluation during PORH

**Figure 4:**
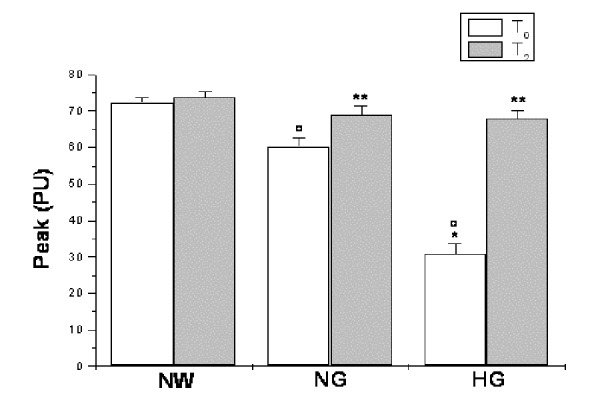
Peak value, expressed as Perfusion Units (PU), under baseline conditions (T_0_) and at six months (T_2_) of diet administration in normal weight (NW), normoglycemic (NG) and hyperglycemic patients (HG). ^∘^ p< 0.01 vs T_0_ NW group, * p< 0.01 vs T_0_ NG group, ** p<0.01 vs T_0_.

**Figure 5:**
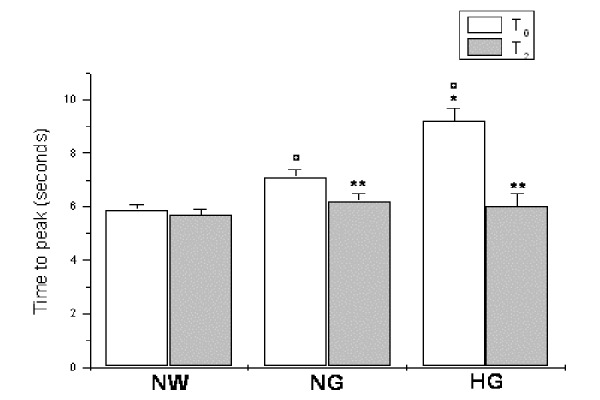
Time to peak, expressed in seconds, under baseline conditions (T_0_) and at six months (T_2_) of diet administration in normal weight (NW), normoglycemic (NG) and hyperglycemic patients (HG). ^∘^ p< 0.01 vs T_0_ NW group, * p< 0.01 vs T_0_ NG group, ** p<0.01 vs T_0_.

Hyperemic response was significantly different in the experimental groups under baseline conditions (time T_0_), resulting impaired in hyperglycemic group. In particular, all groups showed an increase in microvascular blood flow above the baseline following 2 min brachial artery occlusion. However, the peak value was lower in hyperglycemic than in normoglycemic and normal weight subjects (30.8 ± 2.9 vs 60.3 ± 2.5 and 72.3 ± 1.5 PU, respectively; p<0.01 vs NG and NW groups; Figure 4) as well as the percent increase from baseline (345.3 ± 28.0 vs 552.0 ± 27.4 and 670.0 ± 15.6 %, respectively; p<0.01 vs NG and NW groups) and the duration of hyperemia (30.1 ± 1.2 vs 41.9 ± 2.2 and 43.2 ± 1.3 seconds, p<0.01 vs NG and NW groups). Consequently, time to peak was higher in hyperglycemic than in normoglycemic and normal weight subjects (9.2 ± 0.5 vs 7.1 ± 0.3 and 5.9 ± 0.2 seconds, respectively; p<0.01 vs NG and NW groups; Figure 5).

At 3 and 6 months of dietary treatment (time T_1_ and time T_2_, respectively), a significant recovery in hyperemic response was detected in hyperglycemic subjects. At 6 months of treatment, indeed, the peak value significantly increased (68.0 ± 2.1 vs 30.8 ± 2.9 PU, p<0.01 vs T_0_; Figure 4) as well as the percent increase from baseline (729.4 ± 24.9 vs 345.3 ± 28.0, p<0.01 vs T_0_) and the duration of hyperemia (36.4 ± 0.8 vs 30.1 ± 1.2 seconds, p<0.01 vs T_0_). On the other hand, time to peak was significantly reduced in these hyperglycemic patients (6.0 ± 0.5 vs 9.2 ± 0.5 seconds, p<0.01 vs T_0_; Figure 5).

## IV. DISCUSSION

The present results indicate that obesity, in adult patients, is characterized by changes in peripheral microcirculation, under resting conditions as well as during post-occlusive reactive hyperemia (PORH). It is well known that skin microvascular blood flow presents several oscillations due to the different mechanisms regulating the blood flow distribution. Spectral density analysis allowed us to identify the frequency components of these oscillations in the range: 0.005–2.0 Hz. According to previous findings, these frequency components may be related to six different physiological mechanisms [15–17]. Our data show that the sympathetic nervous system discharge and activity of smooth muscle cells are mainly involved in the regulation of microvascular blood flow distribution. However, a significant reduction in the myogenic activity and in NO-independent endothelial activity was observed in the obese subjects, compared to normal weight persons. These changes in the oscillatory patterns were accompanied by a compensatory modification of the high frequency component related to the central drive from the heart, significantly increased in the obese subjects. These data indicate that obesity could be mainly associated to a reduced myogenic activity of arterioles, as previously reported [19]. Moreover, our data indicate that obesity-associated hyperglycaemia represents an additional factor able to exacerbate microvascular impairment. In hyperglycaemic obese patients, indeed, SBF and total power spectral density of skin microvascular blood flow oscillations appeared to be lower than in normoglycemic obese subjects. This finding suggests an increase in peripheral resistance and, consequently, changes in microvascular regulatory mechanisms. Moreover, the myogenic related component appeared significantly reduced, compared to the same component evaluated in normoglycemic obese subjects. These changes were accompanied by a modification of the high frequency component related to respiration, higher in hyperglycemic obese, compared to normoglycemic obese persons. It is worth noting that NO-dependent frequency component, in hyperglycemic obese patients, was not significantly different compared to the same component in normal weight subjects, but higher than in normoglycemic obese persons. Therefore, obesity appears to be characterized by a significant decrease in NO-independent endothelium activity, with a trend to increase in NO-dependent component. Therefore, it is reasonable to hypothesize that hyperglycaemia-induced oxidative stress markedly affects smooth muscle cells, impairing their contractile/relaxation function and increasing peripheral resistance, as indicated by the decrease in NO-independent endothelium activity and further decrease in myogenic activity, with concomitant increase in respiration-related activity [20]. These data are in agreement with previous results, demonstrating severe smooth muscle dysfunction in type II diabetes mellitus [21, 22].

To investigate the responses of skin microcirculation, we used LDPM in combination with post-occlusive reactive hyperaemia (PORH) test. Hyperemic response appeared slight attenuated in obese patients, compared to normal weight persons, and particularly lower in hyperglycaemic obese, compared to normoglycemic obese persons. These data confirm that obesity-associated hyperglycaemia significantly affects microvascular function, under resting conditions as well as after acute hyperaemic response. Since many mediators contribute to the post-occlusive hyperemia, such as axon reflex, different local mediators and vascular smooth muscle cell activity, it is possible to hypothesize that PORH can be related to the overall functions localized in the arteriolar vessel walls [14, 24].

Our results, however, demonstrated that the recovery in nutritional status and the significant reduction in bioumoral parameters, including glycemia, Hb1Ac, basal insulin and HOMA index, were accompanied by an improvement in blood flow oscillations. In particular, the low-glycemic and hypocaloric diet determined a significant increase in SBF and total power spectral density in hyperglycemic obese patients. Similarly, spectral analysis revealed an increase in myogenic activity, indicating an improvement in vascular smooth muscle activity. Accordingly, the high frequency components were differently affected, decreasing the respiration- and increasing the heart rate- related components. Finally, the recovery of smooth muscle cell functions was confirmed by the improvement in the hyperaemic response, observed in these patients.

In conclusion, our data demonstrate that obese subjects have microvascular impairment, exacerbated by hyperglycaemia, mainly inducing a severe disfunction in smooth muscle cell activity with alterations of post-occlusive hyperaemic responses. Therefore, a low-glycemic and hypocaloric diet could promote a recovery of smooth muscle cell functions and microvascular responses.

## Figures and Tables

**Table 1:** Anthropometric measurements [body mass index (BMI), waist circumference (WC), hip circumference (HF) and triceps skinfold (TS)], body composition [Fat Mass (FM) and Fat Free Mass (FFM)] and metabolic parameters [Glycemia (Gly), glycated hemoglobin (HbA1c), basal insulin and HOMA index] under baseline conditions (T_0_), at three (T_1_) and six months (T_2_) of low-glycemic and hypocaloric diet in normoglycemic subjects (NG group).

	NG GROUP					
	FEMALES (n=27)			MALES (n= 27)		
	T0	T1	T2	T0	T1	T2
**BMI**	34.7±0.8	32.9±0.9**	31.6±0.9** ∘∘	34.0±1.1	32.0±1.1**	30.3±0.9** ∘∘
**WC**	105.2±2.1	101.8±2.1**	97.7±1.9** ∘∘	112.9±2.5	108.4±2.5**	101.4±1.6** ∘∘
**HC**	114.3±1.6	111.9±1.6**	108.7±1.5** ∘∘	111.6±2.5	107.0±2.7**	104.8±2.2** ∘
**TS**	28.7±1.4	26.3±1.2**	24.2±1.1** ∘∘	25.4±1.5	22.6±1.6**	20.1±1.4** ∘∘
**FM (Kg)**	37.8±1.6	34.3±1.7**	31.7±1.5** ∘∘	31.5±0.9	25.6±1.7**	21.9±1.5** ∘∘
**FM (%)**	43.5±1.2	41.3±1.4**	39.9±1.0** ∘	31.4±0.9	26.9±0.9**	24.4±1.0** ∘∘
**FFM (Kg)**	48.6±1.1	47.7±0.9	47.0±0.7*	67.7±2.0	67.5±1.7	66.1±1.5*
**FFM (%)**	56.5±1.2	58.7±1.4**	60.1±1.0**	68.6±0.9	73.1±0.9**	75.6±1.0** ∘∘
**Glycemia**	89.9±2.4	89.2±2.5	87.3±2.3* ∘	93.3±1.7	92.8±1.8	90.7±1.9*
**Hb1Ac**	5.5±0.9	5.3±0.5	5.3±0.2	5.7±1.0	5.5±1.3	5.2±0.6
**Basal insulin**	11.2±2.3	9.5±1.4	8.8±1.5	10.9±2.0	9.7±1.5	9.1±1.6
**HOMA index**	2.4±0.4	2.0±0.3	1.7±0.4	2.3±0.3	2.0±0.4	2.0±0.2

Data are reported as mean ± SEM;

* p<0.05 vs T_0_;

** p<0.01 vs T_0_;

∘ p<0.05 vs T_1_;

∘∘ p<0.01 vs T_1_.

**Table 2:** Anthropometric measurements [body mass index (BMI), waist circumference (WC), hip circumference (HF) and triceps skinfold (TS)], body composition [Fat Mass (FM) and Fat Free Mass (FFM)] and metabolic parameters [Glycemia (Gly), glycated hemoglobin (HbA1c), basal insulin and HOMA index] under baseline conditions (T_0_), at three (T_1_) and six months (T_2_) of low-glycemic and hypocaloric diet in hyperglycemic patients (HG group).

	HG GROUP					
	FEMALES (n=27)			MALES (n=27)		
	T0	T1	T2	T0	T1	T2
**BMI**	38.9±1.0	36.6±1.2**	35.4±1.1** ∘∘	37.1±0.8	34.6±0.7**	33.3±0.7** ∘∘
**WC**	110.6±2.1	105.8±1.9**	103.8±1.8** ∘∘	121.3±1.6	114.3±1.5**	110.3±1.6** ∘∘
**HC**	120.1±2.2	116.7±2.5**	115.2±2.3** ∘∘	114.9±1.4	110.3±1.4**	109.6±1.6** ∘
**TS**	34.0±0.8	30.4±1.1**	28.9±1.0** ∘∘	29.2±1.0	23.9±0.7**	22.5±0.8**
**FM (Kg)**	42.9±2.1	39.4±1.7**	37.3±1.6** ∘∘	34.8±1.8	30.9±0.9**	28.2±0.9** ∘∘
**FM (%)**	45.6±1.1	44.7±0.8	43.7±1.1** ∘	31.6±1.0	30.4±0.4	28.9±0.5** ∘∘
**FFM (Kg)**	50.1±1.4	47.9±1.4**	47.3±1.4**	73.6±1.2	70.0±1.4**	69.0±1.5** ∘
**FFM (%)**	54.4±1.1	55.2±0.8**	56.3±1.1** ∘∘	68.4±1.0	69.6±0.4	71.1±0.5** ∘∘
**Glycemia**	121.8±3.2	102.2±1.7**	100.1±1.7** ∘∘	139.5±6.8	115.0±3.5**	104.4±2.2** ∘∘
**Hb1Ac**	7.7±0.5	6.0±0.1**	5.4±0.5	7.1±0.8	5.9±0.1**	5.7±0.1**
**Basal insulin**	15.1±1.5	11.6±1.9**	9.6±2.1∘∘	15.1±1.2	11.5±1.8**	10.7±1.6**
**HOMA index**	4.5±0.4	2.8±0.3**	2.2±0.3**	5.2±0.4	3.1±0.3**	2.6±0.3**

Data are reported as mean ± SEM;

* p<0.05 vs T_0_;

** p<0.01 vs T_0_;

∘ p<0.05 vs T_1_;

∘∘ p<0.01 vs T_1_.

**Table 3:** Anthropometric measurements [body mass index (BMI), waist circumference (WC), hip circumference (HF) and triceps skinfold (TS)], body composition [Fat Mass (FM) and Fat Free Mass (FFM)] and metabolic parameters [Glycemia (Gly), glycated hemoglobin (HbA1c), basal insulin and HOMA index] under baseline conditions (T_0_) and after six months (T_2_) of daily usual diet in normal weigh patients (NW group). Data are reported as mean ± SEM.

	NW GROUP			
	FEMALES (n=27)	FEMALES (n=27)	MALES (n=27)	MALES (n=27)
	T0	T2	T0	T2
**BMI**	23.6±1.0	23.8±1.3	24.5±0.6	25.1±1.3
**WC**	83.0±1.6	83.4±1.0	92.6±1.8	92.3±2.0
**HC**	98.4±1.1	98.1±0.5	95.8±1.5	96.0±1.8
**TS**	17.3±2.2	17.5±1.5	14.3±1.7	14.5±1.5
**FM (Kg)**	20.2±1.3	20.5±2.0	16.3±1.3	16.5±1.8
**FM (%)**	31.4±1.5	31.5±1.8	22.5±1.6	22.7±1.3
**FFM (Kg)**	44.4±2.1	44.5±2.1	55.8±1.4	55.9±2.1
**FFM (%)**	68.5±1.3	68.7±0.6	77.5±1.6	77.7±2.3
**Glycemia**	88±1.6	86.5±2.2	91.3±1.7	91.0±1.5
**Hb1Ac**	5.2±0.5	5.0±0.8	5.1±0.8	4.9±0.5
**Basal insulin**	8.7±1.4	7.7±1.0	9.2±0.9	9.7±1.0
**HOMA index**	1.8±0.4	1.7±0.3	2.1±0.4	2.2±0.6
